# Sources of resistance and susceptibility to Septoria tritici blotch of wheat

**DOI:** 10.1111/mpp.12482

**Published:** 2016-10-20

**Authors:** Lia S. Arraiano, James K. M. Brown

**Affiliations:** ^1^ John Innes Centre, Norwich Research Park Colney Norwich NR4 7UH UK; ^2^ Present address: Vilmorin SA, Centre de Recherche de La Costière Ledenon 30210 France

**Keywords:** association genetics, breeding for disease resistance, Septoria tritici blotch, trade‐offs, wheat, *Zymoseptoria tritici*

## Abstract

An association genetics analysis was conducted to investigate the genetics of resistance to Septoria tritici blotch, caused by the fungus *Zymoseptoria tritici* (alternatively *Mycosphaerella graminicola*), in cultivars and breeding lines of wheat (*Triticum aestivum*) used in the UK between 1860 and 2000. The population was tested with Diversity Array Technology (DArT) and simple‐sequence repeat (SSR or microsatellite) markers. The lines formed a single population with no evidence for subdivision, because there were several common ancestors of large parts of the pedigree. Quantitative trait loci (QTLs) controlling Septoria resistance were postulated on 11 chromosomes, but 38% of variation was not explained by the identified QTLs. Calculation of best linear unbiased predictions (BLUPs) identified lineages of spring and winter wheat carrying different alleles for resistance and susceptibility. Abundant variation in Septoria resistance may be exploited by crossing well‐adapted cultivars in different lineages to achieve transgressive segregation and thus breed for potentially durable quantitative resistance, whereas phenotypic selection for polygenic quantitative resistance should be effective in breeding cultivars with increased resistance. The most potent allele reducing susceptibility to Septoria, on chromosome arm 6AL, was associated with reduced leaf size. Genes which increase susceptibility to Septoria may have been introduced inadvertently into UK wheat breeding programmes from cultivars used to increase yield, rust resistance and eyespot resistance between the 1950s and 1980s. This indicates the need to consider trade‐offs in plant breeding when numerous traits are important and to be cautious about the use of non‐adapted germplasm.

## Introduction

Septoria tritici blotch (‘Septoria’), caused by the fungus *Zymoseptoria tritici* (alternatively *Mycosphaerella graminicola*), first became a major foliar disease of wheat (*Triticum aestivum*) in Europe in the late 1970s (Hardwick *et al*., 2001; Shaw *et al*., 2008). It is especially damaging in humid, temperate areas, such as north‐western Europe (Fones and Gurr, 2015). Although the resistance of wheat cultivars to Septoria has increased over the last two decades (Brown *et al*., 2015), disease control still relies heavily on fungicides. *Zymoseptoria tritici* has adapted to strobilurin and triazole fungicides (Cools and Fraaije, 2012; Torriani *et al*., 2015), and insensitivity to succinate dehydrogenase inhibitors (SDHIs) has been reported recently (AHDB Cereals & Oilseeds, 2016; Teagasc, 2015). This has made Septoria resistance one of the highest priorities in wheat breeding (Brown *et al*., 2015; Torriani *et al*., 2015).

Wheat cultivars vary in the severity of Septoria for two reasons (Brown *et al*., 2015). They may have resistance to *Z. tritici*, reducing infection, growth and reproduction of the fungus. Alternatively, features of plant morphology development may cause disease escape. Increased distance between leaves reduces the rate of spread of spores within the crop, whereas later leaf emergence reduces the time during which the leaf is exposed to infection (Arraiano *et al*., 2009; van Beuningen and Kohli, 1990; Simón *et al*., 2004).

Twenty major *Stb* genes conferring qualitative resistance to Septoria have been identified and mapped (Brown *et al*., 2015). Most are effective only against avirulent genotypes of *Z. tritici*, and resistance can be overcome through the evolution of pathogen virulence (Cowger *et al*., 2000; Krenz *et al*., 2008). More significant in wheat breeding is partial or quantitative resistance (QR), which is incomplete but generally effective against all genotypes of a pathogen, and is usually durable (Brown, 2015; Niks *et al*., 2015). Numerous quantitative trait loci (QTLs) for QR to Septoria have been reported (reviewed by Brown *et al*., 2015; see also Adhikari *et al*., 2015; Dreisigacker *et al*., 2015).

A major gene, *Stb6* on chromosome 3A (Brading *et al*., 2002), has been associated with QR to Septoria in field conditions (Arraiano *et al*., 2009), and QTLs for Septoria resistance have been detected near *Stb6* in several crosses (Brown *et al*., 2015). Disease escape traits also reduced Septoria in field trials (Arraiano *et al*., 2009). In the most resistant cultivars, however, such as Pastiche and Exsept, neither escape traits nor major genes accounted for low Septoria scores (Arraiano *et al*., 2009), implying the presence of effective QR genes.

Association genetics (AG), based on linkage disequilibrium (LD), the non‐random association of alleles at different loci within a population, is a powerful tool to resolve the genetics of complex traits (Ingvarsson and Street, 2011; Korte and Farlow, 2013; Rafalski, 2010). It can locate genes in many cultivars simultaneously, identify numerous alleles and reduce the time required to establish a genetic association (Crossa *et al*., 2007; Ingvarsson and Street, 2011; Yu and Buckler, 2006).

Several AG studies of Septoria resistance have been conducted, involving winter cultivars from continental Europe (Kollers *et al*., 2013), hybrid lines bred from such cultivars (Miedaner *et al*., 2013; Mirdita *et al*., 2015) and an international set of spring cultivars (Gurung *et al*., 2014), as well as a combined linkage and AG analysis of seven biparental crosses (Goudemand *et al*., 2013). In all cases, Septoria resistance was polygenic, controlled by genes distributed over the genome with generally small individual effects. Although some significant QTLs were identified, large proportions of genetic variation were controlled by genes with effects too small to be detected individually. Some QTLs mapped near major *Stb* genes, whereas genes affecting escape traits, such as early heading (*Ppd‐D1*) and semi‐dwarfing (*Rht‐D1b*), were associated with reduced Septoria (Kollers *et al*., 2013). In all of these studies, the sets of cultivars analysed were horizontal in terms of time, consisting of current varieties and first‐generation lines bred from them.

AG requires three sets of data, on plant phenotypes, genetic markers and population structure. In nature, LD has several causes, including random genetic drift, hitch‐hiking selection, epistatic selection and mixing of populations, all of which generate correlations between allele frequencies at unlinked loci (Hedrick, 2011). In plant breeding, intense selection and an extremely high culling rate cause these effects to be stronger than in natural populations (Breseghello and Sorrells, 2006; Rafalski, 2010). Population structure in crop plants can involve complex relationships between pedigrees at several levels and can be represented in two ways: by the classification of genotypes into distinct subpopulations and by a matrix of genetic distances between individuals (Yu *et al*., 2006). Incorporation of population structure in an AG analysis as a random effect in a linear mixed model controls for the genetic background and reduces the frequency of false positive correlations between markers and traits (Vilhjálmsson and Nordborg, 2013).

Here, an AG analysis of Septoria and related disease escape traits was conducted on wheat cultivars from the UK. These are distinct from continental European cultivars, particularly because they have lower vernalization requirements and less cold hardiness (Bonjean and Angus, 2001). The population differed from those used in previous AG studies of Septoria because it was vertical with respect to time, with cultivars covering a 140‐year period from the earliest days of wheat breeding at *c*. 1860 to the end of the 20th century. We identified areas of the wheat genome which are candidates for QTLs for QR to Septoria in naturally infected field conditions, investigated whether different lineages of UK wheat cultivars have different QR genes, and postulated sources of resistance and susceptibility to Septoria in UK wheat breeding. We conclude by discussing the implications of our results for strategies of breeding for durable disease resistance.

## Results

### Phenotypic and genotypic data

Two hundred and twenty‐five wheat lines (Table S1, see Supporting Information) were studied, including cultivars on the Recommended List in the late 1990s (NIAB, 1997−2000) and progenitors to the start of wheat breeding in the 1860s. A few cultivars which were too susceptible to yellow (stripe) rust (*Puccinia striiformis*) to be field trialled for Septoria were excluded. Most lines were winter types.

Simple‐sequence repeat (SSR, microsatellite) markers distributed throughout the genome with approximately five markers per chromosome were scored for 98 lines, including the 24 most susceptible and 24 most resistant lines (Arraiano *et al*., 2009), with 50 further lines linking the pedigrees of these two groups (Table S1). One hundred and twenty‐nine loci were detected by 115 primer pairs (Table S2, see Supporting Information). The fragment size (bp) is given as a suffix to the name of the primer pair. The number of alleles per SSR locus was in the range 2–21, with a mean of 5.52 (Table S2). The number of common alleles, present in five or more cultivars, varied from two to nine, with a mean of 3.28; 7.2% of data were missing, whereas 3.9% of data points represented rare alleles. In addition to 115 informative SSR markers, 13 primer pairs detected monomorphic genotypes (data not shown).

Diversity Array Technology (DArT) identified 525 markers, distributed throughout the genome, as polymorphic among the 225 lines tested, 420 of which were used in AG because of clustering of loci. DArT markers are indicated by the prefix ‘wPt’ followed by the numerical designation used by Triticarte Pty. Ltd. (Canberra, Australia).

### Associations between markers

The LD parameter *r* ^2^, the square of the correlation coefficient of alleles between loci, was not significant for most pairwise comparisons between 80 unlinked SSR and DArT markers. *r* ^2^ varied from 0.000 to 0.4076 with a median of 0.0145. The 95th percentile of the distribution of these estimates, 0.0774, was used as a population‐specific threshold for *r* ^2^ as evidence of linkage (Breseghello and Sorrells, 2006); 14% of linked pairs of loci were in significant LD with a median *r* ^2^ of 0.0539.

**Figure 1:**
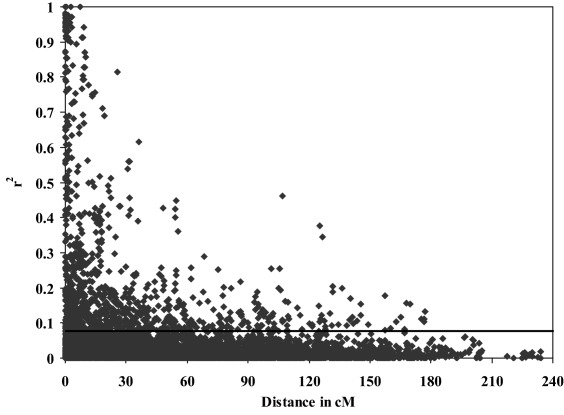
Linkage disequilibrium (*r* ^2^) as a function of map distance for 455 markers, including 340 Diversity Array Technology (DArT) markers and 115 simple‐sequence repeat (SSR) markers, among 225 wheat cultivars. The horizontal line indicates the 95th percentile of the distribution of *r* ^2^ between unlinked markers (*r* ^2^ = 0.0774).

LD declined with increasing map distance between marker loci, with *r* ^2^ falling below the critical value over distances of 30–40 cM (Fig. 1). Blocks of markers in LD (Crossa *et al*., 2007) were identified, the longest being on chromosomes 1B, 7B and 5B, with approximate lengths of 63, 47 and 43 cM, respectively (data not shown).

### Population structure of UK wheat cultivars and breeding lines

Two methods of estimation of the kinship between lines were used, based on pedigree records (pedigree model) and genotypes (*K_T_* model). In the latter model, the kinship matrix *K_T_* was estimated by a method appropriate for autogamous species:

$$K_{Tij}\;=\;\left[\left(S_{ij}-1\right)/\left(1-T\right)\right]+1$$

where *S* is the matrix of genetic similarity and *T* is the probability, averaged over loci, that a pair of lines (*i*, *j*) have alleles which are alike in state, but not identical by descent (Stich *et al*., 2008). Values of *T* from 0 to 0.25 were tested to obtain a residual maximum likelihood (REML) estimate. As the Wald statistics were approximately constant when *K_T_* was estimated with different values of *T*, it was concluded that *T* could be equal to zero, indicating a negligible probability of the same allele having different origins and implying an absence of distinct subpopulations among the lines studied here. The structure program was used to test further the hypothesis of a lack of distinct subpopulations, using the criterion of Evanno *et al*. (2005). Again, no distinct subpopulations were detected.

### Marker associations with morphological traits

Statistical analysis of data on height to flag leaf ligule at maturity (HtFL), heading date (HD), flag leaf length (LL), leaf prostrateness (LP) and logit‐transformed Septoria scored as the percentage diseased area on flag leaves, including correlations between these traits, has been reported previously (Arraiano *et al*., 2009; see also Table S1 for mean scores of lines). AG analysis was conducted on mean scores of lines across trials because, for each trait, the main effect of line was much larger than the line × trial interaction, implying that lines performed fairly consistently in the series of trials (Table 2 in Arraiano *et al*., 2009). Statistically significant associations between traits and markers on 18 chromosomes were detected using the *K_T_* model (Table 1).

**Table 1:** Wald statistics from *K_T_* model for association of simple‐sequence repeat (SSR) and Diversity Array Technology (DArT) loci with height from ground to flag leaf ligule (HtFL), heading date (HD), flag leaf length (LL) and prostrateness (LP), Septoria tritici blotch (STB) and STB adjusted for effect of HtFL (AdjSTB).

Chromosome	Marker*	cM^†^	HtFL	HD	LL	LP	STB	AdjSTB
1A	wPt‐9317	7			5^‡^	**13**		
	wPt‐1167	8	6		**12**	**17**		
	wPt‐6709	8			*10*	**20**		
	*Xgwm136*	12		*30*			*18*	
	wPt‐3870	14			**14**	*9*	6	
	wPt‐1426	57		6	**18**	**21**		
	wPt‐2872	90	**16**		**15**	**14**		
	wPt‐6005	166	**16**		*8*	*8*		
1B	wPt‐1911	42			6	**12**		
	wPt‐2725	47			*9*	**14**		
	*Xbarc8*	49				**23**		
	wPt‐0974	49	5		*9*	**16**		
	wPt‐6117	72		5	5	**17**		
	wPt‐1403	128	5	**15**				
	wPt‐2526	138		**17**				
1D	*Xgwm337*	*47*				**19**		*14*
	wPt‐5320	*49*		*8*		*10*	*11*	**21**
	wPt‐9380	*53*				**12**		*9*
	wPt‐3743	83		**15**				
	wPt‐8545	191					6	*10*
2A	wPt‐6662	170			5	*8*		*9*
2B	*Xwmc154*	66					9	*13*
	wPt‐6278	84		**23**		5		
	*Xbarc167*	158						*10*
	*Xgwm388*	174					8	*12*
	wPt‐1127	182	**14**			8	*9*	
	wPt‐1140	195	5		5	**15**		
	wPt‐5242	241	**11**				6	
	wPt‐7350	247		**12**				
	wPt‐2397	277	5		5	*8*	**12**	6
2D	*Xgwm455*	34	6			**13**	*8*	
	wPt‐2644	126		*10*		**12**		
	wPt‐9997	126					5	*10*
	wPt‐2160	141					**12**	6
	wPt‐2781	147	*8*				**15**	6
	*Xgwm539*	178		14		*16*	14	*19*
	*Xgwm349*	196					11	*17*
3A	*Xbarc12*	20					12	*13*
	wPt‐4407	40				**17**		
	*Xbarc45*	49						9
	wPt‐7608	51					5	*11*
	wPt‐7756	51						*8*
3B	wPt‐1935	3	**26**	**24**			*9*	
	wPt‐7504	3	**26**	**24**			*9*	
	wPt‐7907	3	**28**	**25**			*9*	
	wPt‐1191	70					*7*	
	wPt‐1940	97		5	*7*	**12**		
	wPt‐7502	100	5		*7*	**13**		
	wPt‐1171	140	*7*				*8*	
4A	*Xdupw4*	104					7	*14*
	wPt‐6440	171	**12**					
	wPt‐2151	199	**14**	6				
	wPt‐8091	204	*7*	*8*			*11*	
	wPt‐6688	204	*8*	*10*			*10*	
	wPt‐4620	206		**17**				
4B	wPt‐3991	51		6			*8*	*8*
	wPt‐7062	78						**12**
	*Xbarc163*	83					*11*	*11*
	wPt‐3608	120		**17**				
4D	wPt‐0472	80	**34**	6	*8*	**22**	5	
5B	wPt‐1302	−8			*11*	**13**		
	wPt‐5914	35	6				**12**	
	wPt‐8132	45						*9*
	wPt‐5737	46		5		**14**		
	wPt‐4628	115	**31**		6		*8*	
	wPt‐9613	115	4	**16**			7	
	wPt‐7101	115	**26**		4		5	
	wPt‐3503	116	**26**				*8*	
	wPt‐1250	116	**32**		6	5	*10*	
	wPt‐5896	150					*10*	
5D	wPt‐1400	101	6		*9*	**13**	*11*	6
	wPt‐5505	101	5		*9*	**11**	*9*	
	wPt‐9788	182					**23**	**17**
6A	wPt‐3524	32	**11**			*10*		
	wPt‐3965	37	6	**22**				
	wPt‐3605	38		**14**				
	wPt‐3091	53			6	**13**		
	*Xbarc107*	104	**16**		**13**			
	*Xpsp3029.2*	106	**28**		**31**	**26**	**28**	**21**
	*Xpsp3071*	108			10	**24**	*13*	**25**
	*Xgwm570*	140					10	*17*
	wPt‐8509	207		**13**		6		
6B	wPt‐8894	5	*7*	**16**		5		
	wPt‐4858	24	6	7		**12**		
	wPt‐9594	31				*10*		*7*
	wPt‐2899	34	**15**		5	*11*	7	
	wPt‐4218	34	**15**	*10*			**11**	
	wPt‐5480	145		**13**				
	wPt‐0696	146		**14**				
6D	*Xbarc175*	94		**14**				
7A	wPt‐1928	41		**12**				
	wPt‐6495	193		**21**				
7B	wPt‐5816	173	*11*	**20**			**11**	
	wPt‐7241	175	5	**15**			*8*	
	wPt‐8598	175	5	**16**			*7*	

*Markers listed in this table have *P <* 0.01 for Septoria or AdjSTB or *P* < 0.001 for other traits (*F*‐test).

^†^Map distances are based on the composite map of Maccaferri *et al*. (2015). Details are given in Notes S1 (see Supporting Information).

^‡^ *F*‐test probability of Wald statistic: Roman type, 0.05 < *P* < 0.01; italic type, 0.01 < *P* < 0.001; bold type, *P* < 0.001.

**Table 2:** Wald statistics for the association of simple‐sequence repeat (SSR) and Diversity Array Technology (DArT) loci with Septoria, adjusted for the association with plant height (AdjSTB), shown by single marker associations with no random effect (linear model) or with similarity matrices calculated from the pedigree or markers (*K_T_* model) as random effects.

	Locus			Linear model		Pedigree model		*K_T_* model		Significant in two‐marker model^‡^
Chr.	Marker*	cM^†^	Alleles	Wald	*P*	Wald	*P*	Wald	*P*	
1D	*Xgwm337*	47	4	22.16	<0.001	6.13	0.1	14.29	0.004	
	wPt5320	49	2	19.28	<0.001	10.58	0.001	21.19	<0.001	X
	wPt9380	53	2	9.26	0.003	3.09	0.08	8.56	0.004	
	wPt8545	191	2	10.17	0.002	11.48	<0.001	10.17	0.002	
2A	wPt8132	7	2	9.19	0.003	5.46	0.02	8.63	0.004	X
	wPt2087	7	2	4.03	0.05	7.33	0.007	4.34	0.04	
	wPt7187	13	2	4.04	0.05	7.01	0.009	4.97	0.03	
	wPt6662	170	2	9.08	0.003	12.27	<0.001	8.69	0.004	
2B	*Xwmc154*	66	4	28.71	<0.001	21.27	<0.001	13.07	0.002	X
	*Xbarc167*	158	2	12.24	<0.001	2.05	0.2	9.55	0.002	
	*Xgwm388*	174	3	12.01	0.004	7.95	0.02	11.84	0.004	
2D	wPt9997	126	2	8.70	0.004	3.95	0.05	10.06	0.002	
	*Xgwm539*	178	5	17.89	0.002	7.38	0.1	19.29	0.001	
	*Xgwm349*	196	4	24.28	<0.001	9.46	0.03	16.97	0.002	X
3A	*Xbarc12*	20	4	19.07	<0.001	6.13	0.1	12.62	0.009	X
	*Xgwm369*	31	2	10.88	0.001	3.59	0.06	4.3	0.04	
	*Xbarc45*	49	4	13.58	0.005	8.61	0.04	8.68	0.04	
	wPt7756	51	2	12.06	<0.001	2.07	0.2	8.36	0.004	
	wPt7608	51	2	14.78	<0.001	3.02	0.08	10.71	0.001	
4A	*Xdupw4*	104	4	16.69	0.001	12.96	0.007	14.4	0.004	X
4B	wPt3991	51	2	11.73	<0.001	1.20	0.3	8.06	0.005	
	wPt7062	78	2	14.10	<0.001	1.91	0.2	11.52	<0.001	X
	*Xbarc163*	83	3	18.93	<0.001	7.61	0.03	11.15	0.006	
5B	*Xgwm335*	88	3	5.11	0.08	6.61	0.04	7.16	0.03	
5D	wPt1400	101	2	4.37	0.04	2.72	0.1	5.94	0.02	
	wPt9788	182	2	20.62	<0.001	3.46	0.02	16.72	<0.001	X
6A	*Xpsp3029.1*	27	2	11.05	0.001	3.94	0.05	2.47	0.1	
	*Xpsp3029.2*	106	5	23.40	<0.001	8.79	0.08	20.81	<0.001	
	*Xpsp3071*	108	4	35.85	<0.001	14.48	0.004	25.44	<0.001	X
	*Xgwm570*	140	4	29.60	<0.001	9.95	0.02	17.13	0.002	
6B	wPt9594	31	2	5.64	0.02	4.96	0.03	7.14	0.008	
	*Xgwm626*	98	2	4.66	0.03	5.90	0.02	6.95	0.01	X
7B	*Xbarc72*	49	3	10.75	0.006	4.05	0.1	9.1	0.01	
	wPt2356	136	2	5.13	0.03	4.41	0.04	6.76	0.01	X

*Only markers significantly associated with AdjSTB in the pedigree or *K_T_* models are shown (*P* ≤ 0.05 for SSRs or *P* ≤ 0.01 for DArT markers).

^†^Map distances are based on the composite map of Maccaferri *et al*. (2015). Details are given in Notes S1 (see Supporting Information).

^‡^Combinations of significant markers (*P* < 0.05) for AdjSTB were tested as two‐marker models against single‐marker models by a likelihood ratio test.

#### Plant height

**Figure 2:**
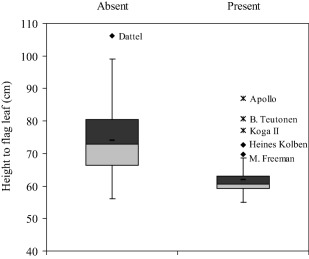
Box‐plots comparing height to flag leaf (HtFL) between lines with or without Diversity Array Technology (DArT) marker wPt‐0472, closely linked to *Rht‐D1b*. Box, interquartile range; horizontal line, median; whiskers, 1.5× interquartile range; filled diamonds, outliers; stars, extreme values >3× interquartile range.

The marker most strongly associated with HtFL was wPt‐0472 on 4D (Table 1, Fig. 2), tightly linked to the semi‐dwarfing gene *Rht‐D1b*, which is widespread in modern cultivars (Srinivasachary *et al*., 2008; it should be noted that *Rht‐B1b* is rare in UK varieties). Markers on chromosomes 1A, 2B, 3B, 4A, 5B, 6A and 6B were also strongly associated with height (*P* < 0.001). QTLs for height have been reported on all these chromosomes (Cadalen *et al*., 1998; Keller *et al*., 1999; Snape *et al*., 2007). The association with chromosome 4A may be related to a potent QTL for lodging in this region (Keller *et al*., 1999) because some of the lines trialled were old, tall wheat varieties with no semi‐dwarfing genes.

#### Heading date

HD was at least moderately associated with all chromosomes, except 7D, and strongly with 12 chromosomes (*P* < 0.001). The strongest association was with markers on chromosome 3B which were also strongly associated with HtFL (Table 1). Numerous genes affecting HD have been reported in wheat and other grasses (Fjellheim *et al*., 2014).

#### Leaf morphology

LL and LP were strongly correlated (Arraiano *et al*., 2009) and, as such, most markers associated with one trait were also associated with the other. Both traits were associated (*P* < 0.01) with chromosomes 1A, 1B, 3B, 4D, 5B, 5D and 6A, and LP only with 1D, 2B, 2D, 3A and 6B. LL and LP are controlled by many minor genes (Habash *et al*., 2007; Nalini *et al*., 2007; ter Steege *et al*., 2005).

#### Associations between traits

**Figure 3:**
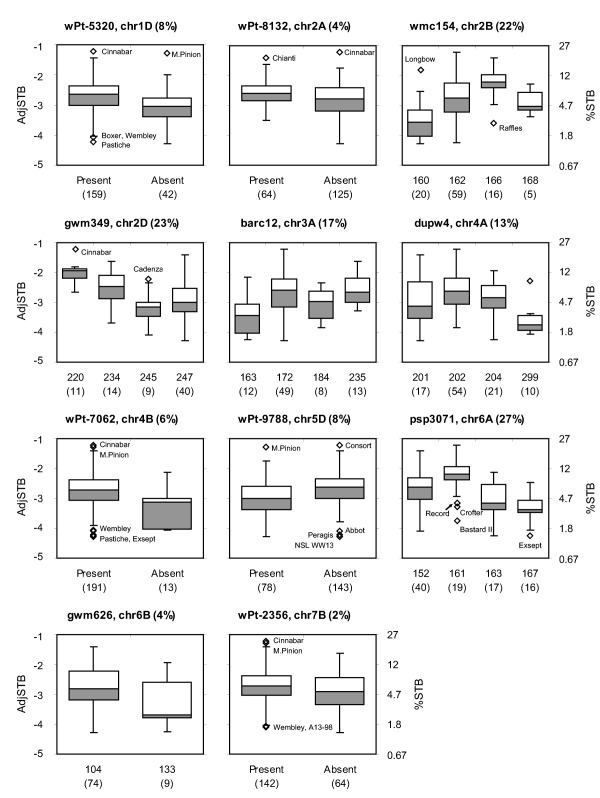
Effects of markers significantly associated with Septoria. AdjSTB data are residuals of logit(Septoria) regressed on height to flag leaf (HtFL); the equivalent percentage leaf area diseased (%STB) is on the secondary axis. Presentation of box‐plots as in Fig. 2. Percentage variation in AdjSTB explained by a general linear model with each marker as the sole explanatory variable is given after the chromosome location. Alleles are fragment sizes (bp) for simple‐sequence repeat (SSR) markers or presence and absence of Diversity Array Technology (DArT) markers, with the number of lines with that allele. Alleles found in fewer than five lines are omitted.

Two closely linked markers on chromosome 6A, *Xpsp3029.2* and *Xpsp3071*, were associated with most of the traits analysed, including logit(Septoria), LL, LP and, in the case of *Xpsp3029.2*, HtFL, but not with HD. Predicted mean LL for the alleles of *Xpsp3071* were 21.3 cm for B152, 23.4 cm for B161, 23.3 cm for B163 and 21.8 cm for B167 (Fig. 3), indicating a 7% reduction in LL between those contributing the highest (B161) and lowest (B167) susceptibility to Septoria (standard error of difference = 0.72 cm, *P* = 0.03). This region of chromosome 6A includes genes controlling yield and yield components (Simmonds *et al*., 2014; Snape *et al*., 2007) which, as complex variables that describe plant productivity, are influenced by many traits, including features of leaf development.

### Association of traits with Septoria

The variate AdjSTB is the mean of logit(Septoria) allowing for plant height as an escape trait, calculated as the residuals from linear regression of predicted mean logit(Septoria) on HtFL (Arraiano *et al*., 2009). Earlier HD has been associated with higher Septoria severity in some studies (van Beuningen and Kohli, 1990; Kollers *et al*., 2013), but, here, that association was weak and inconsistent (Arraiano *et al*., 2009); therefore, AdjSTB did not incorporate variation in HD. Fifty‐two markers were significantly associated with logit(Septoria), but only 21 with AdjSTB. Marker alleles on chromosomes 1A, 3B, 4D, 5B and 7B associated with reduced logit(Septoria), but not AdjSTB, in the *K_T_* model (Table 1) were also associated with increased height, consistent with plant height being a disease escape trait (Arraiano *et al*., 2009).

In an analysis without kinship data, LP and LL were significantly correlated with Septoria (Arraiano *et al*., 2009). When kinship was included in the *K_T_* model, this correlation was no longer present (details not shown).

### Association of markers with Septoria resistance

Associations between markers and AdjSTB were investigated by a simple analysis of variance (ANOVA) model (linear model) and linear mixed models with either the pedigree (pedigree model) or genotypic similarity (*K_T_* model) as a random effect (Table 2). In the linear model, 19% of the 535 markers tested were significantly associated with AdjSTB, but this figure almost certainly included numerous Type I statistical errors. By taking population structure into account, the mixed models reduced the number of markers associated with AdjSTB. Marker–trait associations are therefore only considered for the mixed models. The pedigree and *K_T_* models were generally, but not completely, consistent in the markers reported as significantly associated with AdjSTB.

AdjSTB was associated with 11 of the 21 chromosomes. There was strong evidence (*P* < 0.001 with either method) for associations with chromosomes 1D, 2A, 2B, 4B, 5D and 6A, and moderate evidence (*P* < 0.01) for associations with chromosomes 2D, 3A, 4A, 6B and 7B (Table 2). Combinations of markers significantly associated with AdjSTB (*P* < 0.05) were tested as two‐marker models against single‐marker models by likelihood ratio tests. Only a few markers on each chromosome remained significant, consistent with linked markers being in LD and with there being one QTL on each chromosome. The marker on each chromosome most strongly associated with AdjSTB following two‐marker tests is labelled ‘X’ in Table 2. An additive model with all 11 of these focal markers explained 62% of the genetic variation in AdjSTB. The strongest effect was that of *Xpsp3071* on chromosome 6A, explaining 27% of the variation.

Allelic effects and percentage variance explained were calculated for the 11 focal markers (Fig. 3). Flame and NSL 94‐5130 had Septoria‐reducing alleles of five of the six markers most significantly linked to AdjSTB, whereas Exsept, Claire and Arina had four (Table S1). All these lines, except Arina, were bred by Nickerson UK Ltd., (Market Rasen, UK) now part of Limagrain UK Ltd., and all but Claire were among the top 20 most resistant to Septoria in field trials (table 7 in Arraiano *et al*., 2009).

**Figure 4:**
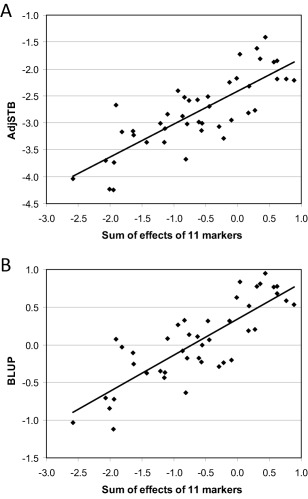
Correlation of AdjSTB with the sum of predicted effects on AdjSTB of 11 focal markers (Fig. 3) for 43 cultivars and breeding lines which were scored for all 11 markers and had no rare alleles. (A) Relationship of predicted mean AdjSTB in field trials to AdjSTB predicted from 11 focal markers; correlation coefficient (*r*) = 0.80. (B) Relationship of best linear unbiased prediction (BLUP) of AdjSTB to AdjSTB predicted from 11 markers; *r* = 0.82.

For 43 lines, there were data on all 11 focal markers (Table 2) with no rare alleles present in fewer than five lines. Among these lines, there was a strong correlation between the sum of predicted allelic effects on the one hand, and both observed AdjSTB (*r* = 0.80) and the best linear unbiased predictor (BLUP) of the breeding values of lines (*r* = 0.82; Fig. 4) on the other.

### Breeding values for Septoria resistance

Breeding values of AdjSTB for the 226 lines field trialled were estimated as BLUP scores from a linear model with line fitted as a random effect, the covariance structure estimated from the pedigree model and no fixed effect (Table S1). BLUP scores of AdjSTB were also calculated for 366 progenitor lines which were not field trialled. Calculation of BLUP allows the identification of lineages which have contributed resistance or susceptibility to breeding programmes, including lines which were not field trialled themselves.

### Sources of resistance and susceptibility to Septoria

**Figure 5:**
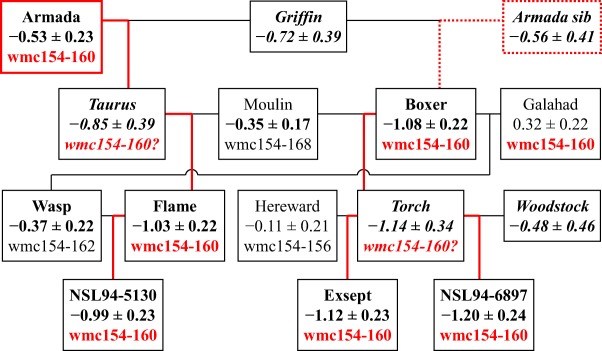
Inheritance of Septoria resistance and allele *Xwmc154‐160* on chromosome 2B in cultivars bred from cv. Armada and a sibling in the 1980s and 1990s. Red lines: inheritance of *Xwmc154‐160* from Armada and tentatively Armada sib. Data are values and standard errors of the best linear unbiased predictor (BLUP) of AdjSTB. Band sizes (bp) of amplified DNA fragments follow the locus name. Cultivar name and BLUP data in bold: BLUP lower than one standard deviation of BLUP across all lines (0.32), implying that the line has quantitative resistance to Septoria. Data in italics: cultivar not field trialled, with marker allele, where shown, inferred from progeny data.

The six winter wheats with the lowest BLUP of AdjSTB (i.e. the best breeding values for Septoria resistance) were a group of lines bred by Nickerson UK Ltd. from several moderately resistant progenitors, including Armada and a sibling line, Griffin, Moulin and Woodstock. A QTL linked to *Xwmc154‐160* on chromosome 2B, present in all resistant cultivars in this lineage, may have been introduced from Armada and its sibling (Fig. 5).

**Figure 6:**
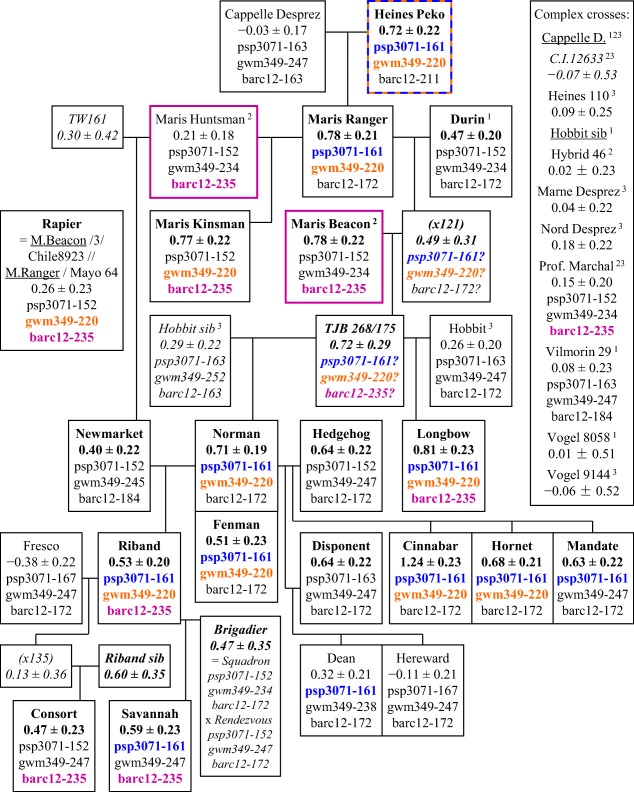
Inheritance of Septoria susceptibility in cultivars bred from Heines Peko and Cappelle Desprez and released from the 1960s to the 1990s. The inheritance of allele *Xpsp3071‐161* (chromosome 6A) from Heines Peko is indicated in blue type, *Xgwm349‐220* (chromosome 2D) also from Heines Peko in orange, and *Xbarc12‐235* (chromosome 3A) from Maris Huntsman and Maris Beacon (and tentatively, from their grandparent Professeur Marchal) in purple. Superscript 1, 2, 3: parentage of Durin, Maris Huntsman and Maris Ranger, and Hobbit respectively. For further details of presentation, see Fig. 5.

Three alleles contributed particularly to a Septoria susceptibility in a lineage of cultivars bred by the Plant Breeding Institute (PBI, Cambridge, UK) between the mid‐1960s and the mid‐1980s (Fig. 6). Two of the most potent alleles, linked to *Xpsp3071‐161* on chromosome 6A and *Xgwm349‐220* on chromosome 2D, almost certainly entered UK breeding from Heines Peko via Maris Ranger (H.Peko × Cappelle Desprez), because it is the most recent common progenitor of cultivars known to have these alleles. Norman was an especially important link in this lineage. The third gene, linked to *Xbarc12‐235* on chromosome 3A, probably came from Professeur Marchal via the sibling lines Maris Huntsman and Maris Beacon.

**Figure 7:**
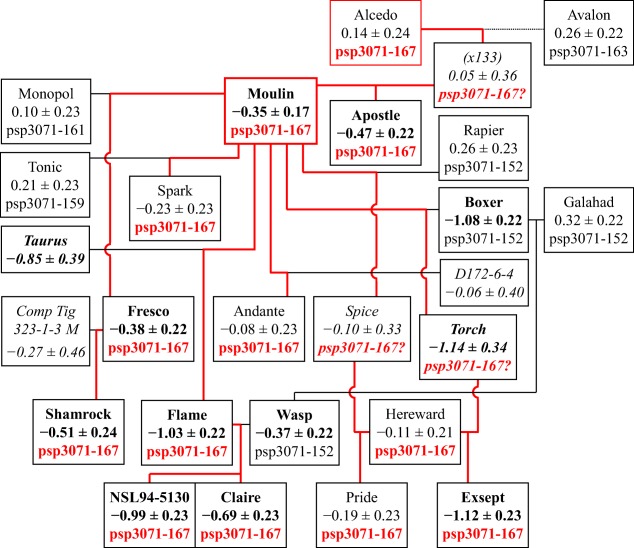
Inheritance of Septoria resistance and the allele *Xpsp3071‐167* on chromosome 6A in cultivars bred from Moulin in the 1980s and 1990s. Red lines: inheritance of *Xpsp3071‐167* from Moulin. For further details of presentation, see Fig. 5.

On the other hand, Moulin, also bred by PBI, contributed one of the most potent alleles increasing resistance, linked to *Xpsp3071‐167* (Fig. 7). The combination of this allele and *Xwmc154‐160* accounts for a substantial proportion of the resistance of lines, such as Exsept, Flame and NSL94‐5130.

**Figure 8:**
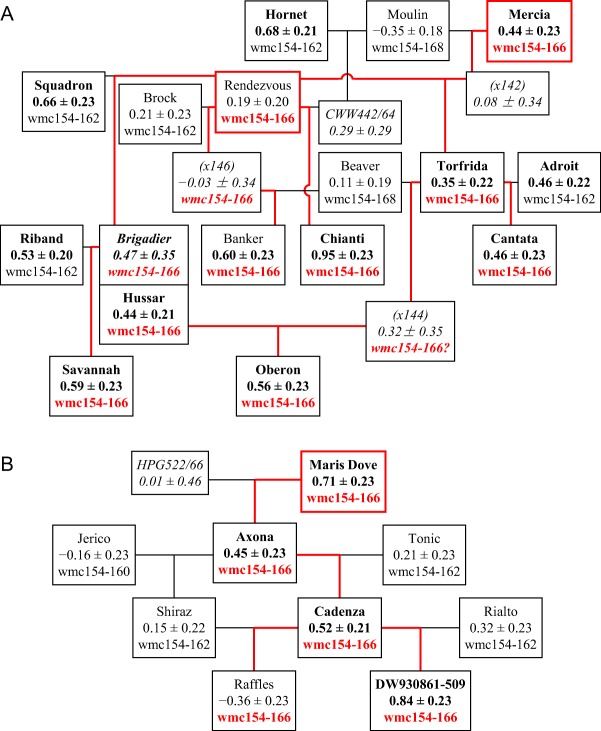
Inheritance of Septoria susceptibility and the allele *Xwmc154‐166* on chromosome 2B in two lineages of cultivars bred in the 1980s and 1990s. (A) Winter cultivars bred from Rendezvous; Torfrida, Adroit, Cantata and Oberon may also have inherited *Xwmc154‐166* from Mercia. (B) Spring cultivars bred from Maris Dove. Red lines: possible paths of inheritance of *Xwmc154‐166*. For further details of presentation, see Fig. 5.

*Xwmc154‐166* was associated with susceptibility in several winter cultivars (Fig. 8A). All those with this allele were descendants of Rendezvous, apart from Mercia, both of which were bred by PBI. Both cultivars were progenitors of Cantata, Oberon and Torfrida. An allele of the same size was transmitted from Maris Dove to a group of spring cultivars (Fig. 8B), but the relationship of the genes in the three sources of *Xwmc154‐166* is not known.

A group of resistant spring wheats bred by PBI from Sicco × Sandown in the 1980s had low BLUP of AdjSTB, including Wembley (−0.92), Anduril (−0.81), Haydock (−0.73) and Solitaire (−0.48). All four cultivars had the allele *Xdupw4‐299* on chromosome 4A, in common with their apparently more susceptible parent, Sicco (0.13). Their other parent, Sandown (estimated BLUP = −0.94), was not field trialled for Septoria because it was extremely susceptible to yellow rust.

Other winter wheats of diverse origins had notably low BLUP, indicating high breeding values for Septoria resistance. They included A13‐98, Arina, Pastiche and its parent Jena, Riebesel 57/41 (the donor of the 1BS‐1RL wheat–rye translocation), Bastard II, Greif, Arminda and its parent Ibis, Epoch, and Shamrock.

## Discussion

### Population structure of UK wheat cultivars

Several QTLs associated with Septoria severity were identified in UK wheat lines released between *c*. 1860 and 1999 (Table 2, Fig. 3). The close relationship between these lines, often through several links in the pedigree (Table S1), had three consequences for AG analysis. First, there were no identifiable subpopulations, and so the inheritance of genes affecting Septoria was studied in what was effectively a single population, albeit one covering several generations. This facilitated the detection of QTLs. Second, LD halved over a distance of 30–40 cM in this population (Fig. 1), similar to the distance over which LD decayed in CIMMYT breeding lines (Crossa *et al*., 2007), but longer than in a set of cultivars from several European countries (Würschum *et al*., 2013) and much longer than in North American cultivars selected to represent the full range of local diversity (Chao *et al*., 2010). Decay of LD was slow over the timescale of the population studied here because there had not been sufficient time for much recombination to occur between genes that were closely linked in founder genotypes. This reduced the precision with which QTLs could be mapped. Third, it permitted comparisons between two methods of estimating kinship, from pedigree data or genetic similarity. If pedigree information is reasonably complete, the two methods produce similar results (Zhang *et al*., 2006), as happened here (Table 2). The strong association between HtFL and wPt‐0472 on chromosome 4D, which is tightly linked to *Rht‐D1b* (Fig. 2), was a positive control for the methodology used here.

### Genetic structure of Septoria resistance

As in previous work (Brown *et al*., 2015), QR to Septoria in UK wheats was controlled by numerous genes with moderate to small effects, with 11 QTLs detected on as many chromosomes (Table 2, Fig. 3). Together, they accounted for the substantial majority of genetic variation in QR to Septoria (Fig. 4). Larger populations would be required to detect additional QTLs with smaller effects.

Map locations of QTLs discovered by AG in crop species should be regarded as provisional and as subject to validation in controlled crosses, because some important assumptions underlying AG are not met either in this population or in other cultivars of inbreeding plants. It is assumed that LD, on which AG methods are based, declines with genetic map distance, but the extremely intense selection and repeated bottlenecks in breeding programmes generate LD which does not necessarily reflect genetic linkage. The incorporation of a kinship matrix in a mixed model partially corrects for the effects of population structure (Vilhjálmsson and Nordborg, 2013), but this is more effective in large, outbreeding populations than in moderate‐sized populations of an inbreeding species, such as that used here.

In general, there are two responses to the challenge of accounting for population structure in AG. One is to use a conservative procedure for the identification of QTLs to minimize the number of false positive reports. Here, a more liberal approach was taken, accepting the possibility of some Type I errors in order to avoid false negatives (Type II errors), when a real QTL is not observed. The choice is a matter of judgement. Our view is that, given that some important assumptions underlying AG methods do not conform to the realities of plant breeding, it is useful to report QTLs which are likely to affect Septoria as a starting point for further genetic analysis, for example in relevant biparental crosses.

The most important QTL reported here was at a location not reported previously, on chromosome arm 6AL (Table 2). QTLs have been identified in biparental crosses on chromosome arms 1DS (Simón *et al*., 2004), 3AS (Eriksen *et al*., 2003; Kelm *et al*., 2012; Tabib Ghaffary *et al*., 2011; Zwart *et al*., 2010), 4AL (Kelm *et al*., 2012; Risser *et al*., 2011) and 6BL (Chartrain *et al*., 2004; Eriksen *et al*., 2003). In a meta‐analysis of several crosses, QTLs were identified on 2AS, 2BS, 2DL, 3AS and 4AL (Goudemand *et al*., 2013). Previous AG studies of European wheat cultivars have detected Septoria QTLs on chromosome arms 2DL, 5DS, 6BL, 7BS (Kollers *et al*., 2013), 3AS, 4BS (Goudemand *et al*., 2013; Kollers *et al*., 2013) and 4AL (Goudemand *et al*., 2013). The diverse types of marker, mapping methods and populations used in wheat genetics make it difficult to determine whether QTLs identified at the same location in different studies are indeed the same, although comparative maps are available (Maccaferri *et al*., 2015).

The population studied here has been used previously to test the effects of five major genes, *Stb6*, *Stb9*, *Stb10*, *Stb12* and *Stb15*, on Septoria in naturally infected field trials, of which only *Stb6* on 3AS was associated with a significant reduction (Arraiano *et al*., 2009). *Xbarc12* (Table 2, Fig. 3) is closely linked to markers near *Stb6* (Eriksen *et al*., 2003), most of the cultivars used in early breeding for resistance carried *Stb6* (Chartrain *et al*., 2005b), and the *Stb6* region has been identified repeatedly as containing a Septoria QTL (Fig. 3; Eriksen *et al*., 2003; Goudemand *et al*., 2013; Kelm *et al*., 2012; Kollers *et al*., 2013; Tabib Ghaffary *et al*., 2011; Zwart *et al*., 2010). It is not known, however, whether *Stb6* itself contributes to field resistance. *Xwmc154* is close to *Stb9* (Chartrain *et al*., 2009), whereas wPt‐5320 (Yu *et al*., 2014) is close to *Stb10* on chromosome 1D (Chartrain *et al*., 2005a), but there was no evidence that *Stb9*, found in only a few cultivars, and *Stb10*, which was not detected in any of these cultivars (Arraiano and Brown, 2006), contributed to field resistance (Arraiano *et al*., 2009). *Xpsp3071* is on chromosome arm 6AL, whereas *Stb15* on 6AS (Arraiano *et al*., 2007) is present in 60% of these cultivars (Arraiano and Brown, 2006) and was not associated with Septoria levels (Arraiano *et al*., 2009).

AG helped to determine relationships between developmental traits and Septoria severity. It confirmed that plant height (HtFL) is associated with disease escape (Arraiano *et al*., 2009), as in previous studies (Baltazar *et al*., 1990; van Beuningen and Kohli, 1990; Kollers *et al*., 2013; Miedaner *et al*., 2012; Simón *et al*., 2004), but indicated that the association of LL and LP with Septoria (Arraiano *et al*., 2009) was a chance effect, as they were not correlated significantly with Septoria in the *K_T_* mixed model. Nevertheless, there was an intriguing association between marker *Xpsp3071* and both Septoria and leaf morphology, with allele *Xpsp3071‐167* contributing shorter LL and lower Septoria. Smaller leaves may contribute to disease escape because they can sustain fewer lesions.

### Sources of genes affecting Septoria

The highly connected pedigree allowed the history of susceptibility and resistance to Septoria to be investigated. It has been suggested that the emergence of Septoria as a serious problem in the 1970s was stimulated by the use of semi‐dwarfing genes (Baltazar *et al*., 1990), but some cultivars released before 1970 had high foliar susceptibility to Septoria, notably Peragis, Holdfast, Heines Peko, Maris Ranger, Maris Beacon and Maris Kinsman (Table S2), none of which were semi‐dwarf. Genes increasing Septoria susceptibility were therefore present in UK wheat germplasm before the era of semi‐dwarf cultivars, including those linked to *Xpsp3071‐161* on chromosome 6A and *Xgwm349‐220* on 2D introduced from Heines Peko, and *Xbarc12‐235* on 3A, which probably came from Professeur Marchal via Maris Huntsman and Maris Beacon (Fig. 6).

During the 1980s and 1990s, the first and last of these alleles were partially replaced by *Xpsp3071‐167* from Moulin (Fig. 7) and *Xbarc12‐163* in diverse lineages, including Boxer, Flame, Wasp and NSL94‐5130, bred by Nickerson, and Pastiche and Hereward, bred by PBI (Table S1). *Xbarc12‐163* is also widespread in older germplasm, including Jena (a parent of Pastiche), Cappelle Desprez and its parent Hybride du Joncquois, Arina and also, remarkably enough, Peragis, which had high foliar susceptibility and was a parent of Heines Peko (Table S1). The repeated identification of chromosome arm 3AS as a locus for Septoria QR (Brown *et al*., 2015) might relate to genes linked to *Xbarc12‐163*.

During the 1980s, two alleles linked to *Xwmc154* on chromosome 2B with contrasting effects were introduced. *Xwmc154‐166*, linked to susceptibility, came from Rendezvous, Mercia and Maris Dove (Fig. 8). *Xwmc154‐160*, associated with resistance, entered modern germplasm from Armada or its sibling (Fig. 5), but this may have been a reintroduction because it was also present in some older cultivars, notably Cappelle Desprez (Table S1).

Another allele strongly associated with resistance was *Xgwm626‐133* on chromosome 6B in Pastiche and its parent Jena, Arina, Haydock and Epoch (Table S1). These cultivars are not known to be closely related.

The use of a historic set of cultivars can identify lineages which are not closely related to the main body of germplasm, but may be exploited to advance a trait. An example is a group of four spring cultivars, Wembley, Anduril, Haydock and Solitaire, all bred from Sicco × Sandown, which carried the strongest QTL for resistance, linked to *Xdupw4‐299* on chromosome 4A (Fig. 3).

QTLs on five chromosomes were associated with DArT markers, rather than SSRs, but we have not attempted to trace their inheritance. Differential intensity of DArT hybridization can arise from several molecular variants (Jaccoud *et al*., 2001), implying that one should not assume that the presence or absence of a particular array feature represents a single allele.

### Applications to resistance breeding

Breeders have advanced Septoria resistance by selecting for higher QR, which has been durable (Brown, 2015; Brown *et al*., 2015; Torriani *et al*., 2015). Further advances in durable resistance are required as options for the use of fungicides to control Septoria diminish (Torriani *et al*., 2015). The elimination of highly susceptible cultivars advances durable resistance because they not only suffer severe disease themselves, but may transmit spores to other, less susceptible cultivars (Brown, 2015). An especially useful application of AG to plant breeding is to identify lineages which have different genes controlling a trait. Lines from different lineages can then be crossed to achieve transgressive segregation and advance the trait in new cultivars.

A marker‐based strategy for the reduction of Septoria susceptibility would involve selection against alleles *Xwmc154‐166* (Fig. 8), *Xbarc12‐235*, *Xpsp3071‐161* (Fig. 6) and *Xgwm349‐220*. Among the 53 lines with none of these alleles, the mean BLUP value was −0.19, whereas 32 had negative BLUP, indicating better than average resistance (Table S1). Nine such lines had BLUP lower than two standard deviations below zero: Exsept, Boxer, Flame, NSL94‐5130, Wembley, Haydock, Pastiche, Jena and Arminda. QR could be enhanced by selecting for *Xwmc154‐160* (Fig. 5), *Xpsp3071‐167* (Fig. 7) and *Xbarc12‐163*. Two lines with all three of these alleles, Flame (BLUP = −1.03) and NSL94‐5130 (BLUP = −0.99), were among the most resistant of those tested. *Xdupw4‐299*, *Xgwm626‐133* and *Xgwm349* alleles *245* and *247* may also contribute to resistance.

It is striking that, as in previous AG studies of Septoria, a substantial proportion of variation could not be attributed to detectable QTLs. The genes that control this fraction of QR presumably have smaller effects on Septoria resistance. Improved methods of phenotypic selection against high susceptibility would advance durable resistance by promoting the accumulation of numerous genes of small and moderate effects (Brown, 2015).

### Trade‐offs of Septoria resistance

It has often been proposed that sources outside the gene pool of cultivated wheat may be required to increase Septoria resistance in elite wheat cultivars (Arraiano *et al*., 2001; Jing *et al*., 2008; Naz *et al*., 2015; Simón *et al*., 2010; Tabib Ghaffary *et al*., 2011). The data presented here support an alternative view, that combinations of QR genes in well‐adapted germplasm (Figs 3 and 5−8) can advance durable resistance to Septoria (Fig. 4). The history of Septoria resistance in the UK illustrates the potential hazards of using non‐adapted cultivars to improve individual traits.

First, the German cultivar Heines Peko was used in the mid‐1950s by PBI to improve yield and yellow rust resistance (Lupton and Bingham, 1960). Although its gene *Yr6* for yellow rust resistance was rapidly overcome by virulent *P. striiformis* (Johnson, 1992), it also seems to have contributed genes which increased susceptibility to Septoria, replacing alleles which promoted resistance in important older cultivars, such as Cappelle Desprez (Fig. 6). A short‐term benefit in rust control may therefore have come at the expense of an unforeseen, long‐lasting increase in susceptibility of the UK wheat crop to Septoria. The 6A allele from Heines Peko linked to *Xpsp3071‐161* is especially interesting because it is closely linked to genes for increased leaf size (Table 1) and for increased yield in Rialto, Savannah and Badger (Simmonds *et al*., 2014; Snape *et al*., 2007). This may have sustained its use in UK wheat breeding, despite its detrimental effect on Septoria control. Selection against this allele to reduce susceptibility to Septoria may entail a lower yield potential.

Second, Rendezvous was the first cultivar released with *Pch1* for resistance to eyespot (*Oculimacula* spp.) on a segment of chromosome 7D introgressed from *Aegilops ventricosa* via VPM1 (Worland *et al*., 1988). It also contributed genes for Septoria susceptibility linked to *Xwmc154‐166* (Fig. 8), replacing *Xwmc154‐160* in cultivars such as Cappelle Desprez.

Third, by contrast, Moulin was not commercially successful because it suffered from male sterility in trials in 1985 (Law, 2000), but it became an important parent in UK wheat breeding. Part of its value may have been its combination of genes affecting Septoria, because it lacks all four of the main SSR alleles associated with susceptibility and has alleles of *Xwmc154* and *Xpsp3071* associated with resistance.

The benefits of using exotic lines may outweigh the potential costs, but breeders should be aware that, in using such germplasm, they may introduce deleterious traits alongside desirable genes, and that it may take many years to mitigate these undesirable effects. This seems to have happened in the cases of Heines Peko, Rendezvous and Moulin.

## Experimental Procedures

### Phenotypic data

Data on HtFL, HD, LL, LP and logit(Septoria) caused by natural infection by *Z. tritici* were predicted means for 226 lines in 12 field trials over 3 years at sites in eastern England (Arraiano *et al*., 2009). Postulated *Stb* genes and isolate‐specific resistances in these lines were reported by Arraiano and Brown (2006).

### DArT and SSR markers

Genomic DNA of 225 lines (226 which were field trialled, less Cleo) was extracted from 8‐day‐old seedlings using the DNeasy® 96 Plant Kit (Qiagen, Manchester, UK). DArT genotyping of all 225 lines was performed by Triticarte Pty. Ltd. Hybridization of genomic DNA to the *Pst*I(*Taq*I) Version 2.3 DArT array of 2500 clones, image analysis and polymorphism scoring followed Akbari *et al*. (2006). Although DArT markers are biallelic with presence dominant over absence, wheat cultivars are strongly selfing and thus homozygous at most loci. Marker quality was estimated as a percentage *P* value (0–100), reflecting separation of the two phases of the marker. Data from markers with *P* > 80 were used here. Unreliable DArT phenotypes were scored as missing.

SSR markers (Eujayl *et al*., 2002; Pestsova *et al*., 2000; Röder *et al*., 1998; Somers *et al*., 2004; Song *et al*., 2005; Stephenson *et al*., 1998) were studied in 98 lines. Polymerase chain reaction (PCR) was performed using HotStarTaq Master Mix (Qiagen) using fluorescent primers labelled with 6‐FAM, NED, PET, VIC or HEX dye labels (Applied Biosystems, Waltham, USA). PCR products were separated on an ABI3700 sequencer and their sizes were measured using Applied Biosystems’ GeneScan and Genotyper fragment analysis software. Some primers amplified more than one fragment, in which case each was scored as a separate locus. All lines were treated as homozygous, but some mixture of alleles was observed, in which case the more common allele was retained in the analysis, reflecting the more common genotype (following Breseghello and Sorrells, 2006).

Marker alleles present in fewer than five lines were pooled with missing data or null alleles in the AG analysis, because rare alleles inflate the calculated value of *r* ^2^ (Somers *et al*., 2007).

### Population structure

In the pedigree model, kinship was calculated from the coefficient of parentage using data from the European Wheat Database (http://genbank.vurv.cz/ewdb/), the Germplasm Resource Information Program for Wheat (http://www.plantsci.missouri.edu/grip/) and the breeding companies which supported this project (Table S1). Coefficients of parentage were estimated using the VPEDIGREE directive in GenStat (VSN International, Hemel Hempstead, Hertfordshire, UK) to generate a sparse inverse relationship matrix.

In the *K_T_* model, kinship was estimated from the genetic similarity of 42 SSR and 38 DArT markers at unlinked or distantly linked loci distributed across the wheat genome. structure (Pritchard *et al*., 2000) was used to test the hypothesis of one to ten subpopulations (*U*), with or without admixture and with correlated allele frequencies. The burn‐in time for the Markov Chain Monte Carlo algorithm was 100 000 for each run. Ten independent runs were made for *U* = 1 to *U* = 10, and the average likelihood across these runs was calculated for each *U*. The *K_T_* matrix of genetic distances was estimated from marker data by a REML method (Stich *et al*., 2008) using GenStat. AG analysis of SSR markers for the subset of 98 lines was performed using *K_T_* calculated from 80 unlinked SSR and DArT markers. AG analysis of DArT markers used *K_T_* calculated from only the 38 unlinked DArT markers, because the similarity matrix calculated from all 80 markers for all 225 lines was ill‐conditioned.

TASSEL (http://www.maizegenetics.net) was used to calculate the LD parameter *r* ^2^ and its significance for pairs of SSR and DArT loci on the same chromosome. Comparison‐wise significance was computed with 1000 permutations. *r* ^2^ was calculated for all 225 lines with 416 DArT markers, for 98 lines tested with SSR markers, and for 80 unlinked SSR and DArT markers used to create the similarity matrix, *K_T_*.

### Association analysis

The REML directive in GenStat was used to fit the linear, pedigree and *K_T_* models. In mixed models, the variance structure for random effects was calculated by the directive VSTRUCTURE using the inverse relationship matrix produced by VPEDIGREE or the *K_T_* kinship matrix estimated from unlinked markers. Marker–trait associations were assessed by means of Wald and *F* statistics, and classified according to the *P* value of both statistics as strong (*P* < 0.001), moderate (0.001 < *P* < 0.01), weak (0.01 < *P* < 0.05) or not significant (*P* > 0.05).

## Authors’ Contributions

LSA and JKMB planned the research, performed the analysis and wrote the paper. LSA obtained the marker data.

## Supporting information

Additional Supporting Information may be found in the online version of this article at the publisher's web‐site:

**Note S1** Positions of loci in Tables 1 and 2.

**Table S1** Data on phenotypes, markers and pedigrees of 226 wheat cultivars and breeding lines field trialled for susceptibility to Septoria tritici blotch (*Zymoseptoria tritici*) with best linear unbiased predictions of susceptibility of a further 368 progenitor lines.

**Table S2** One hundred and twenty‐nine simple‐sequence repeat (SSR) loci tested, with estimated position and number of alleles detected and loci chosen for analysis of population structure.
