# Supplementary material for: Sources of resistance and susceptibility to Septoria tritici blotch of wheat
Source: Mol Plant Pathol. 2016 Oct 20;18(2):276–92. doi: 10.1111/mpp.12482 (PMC5297993; doi:10.1111/mpp.12482)
Supplement: Supplementary file 1 — Note S1 Positions of loci in Tables 1 and 2. [file MPP-18-276-s001.pdf]

L.S Arraiano & J.K.M. Brown, 2016. Sources of resistance and susceptibility to Septoria tritici blotch of wheat. ***Molecular Plant Pathology*****. DOI : 10.1111/mpp.12482.**

Supporting Information, Note S1

Positions of loci in Tables 1 and 2

The locations of markers in Table 1 are based on File S4 of Maccaferri *et al.* (2015) with the following exceptions, which were not included in that map.

Chromosome 1B
wPt-0974 was mapped to the same locus as *Xbarc8* (Lowe *et al.*, 2011).

Chromosome 1D
wPt-8545 was mapped to the same locus as wPt-6560 and wPt-4988 (Lowe *et al.*, 2011), which were mapped at 189 and 193 cM respectively by Maccaferri *et al.* (2015).

Chromosome 2A
wPt-8132 was mapped to the same locus as wPt-9839 (Cuthbert, 2011), which was mapped at 7cM by Maccaferri *et al.* (2015)

Chromosome 2D
wPt-9997 was mapped very close to wPt-2644 (Marone *et al.*, 2013).

Chromosome 3B
wPt-7907 was mapped to the same locus as wPt-1935 and wPt-7504 (Lowe *et al.*, 2011).

Chromosome 4D
wPt-0472 was mapped 6cM from *Xpsp3103* (Srinivasachary *et al.*, 2008; this map appears to be inverted with reference to that of Maccaferri *et al.*, 2015). *Xpsp3103* was mapped to the same locus as *Xgwm165* (Komugi composite wheat map) and *Xgwm165* was mapped at 86cM by Maccaferri *et al.* (2015).

Chromosome 5B
wPt-1302 was mapped 54cM above wPt-5737 (Yu *et al.*, 2014).

Chromosome 6A
Xpsp3029.2 was mapped 2cM above *Xpsr915* (GrainGenes Ta-Gale-6A map), which was mapped at 108cM by Maccaferri *et al.* (2015).

Xpsp3071 mapped to the same locus as *Xpsr915*.

wPt8509 was mapped 1cM below *Xgwm427* (Wang *et al.*, 2012), which was mapped at 206cM by Maccaferri *et al.* (2015).

Xpsp3029.1 was mapped 2cM above *Xpsr8(Cp3)* (GrainGenes Ta-Gale-6A map), which was mapped at 29cM by Maccaferri *et al.* (2015).

REFERENCES

Cuthbert, R.D. (2011) Molecular mapping of Septoria tritici blotch resistance in hexaploid wheat (*Triticum aestivum* L.). Ph.D. thesis, University of Manitoba.

Lowe, I., Jankuloski, L., Chao, S., Chen, X., See, D. and Dubcovsky J. (2011) Mapping and validation of QTL which confer partial resistance to broadly virulent post-2000 North American races of stripe rust in hexaploid wheat. *Theor. Appl. Genet.* **123,** 143-157.

Maccaferri, M., Zhang, J., Bulli, P., Abate, Z., Chao, S., Cantu, D., Bossolini, E., Chen, X., Pumphrey, M. and Dubcovsky, J. (2015) A genome-wide association study of resistance to stripe rust (*Puccinia striiformis* f.sp. *tritici*) in a worldwide collection of hexaploid spring wheat (*Triticum aestivum* L.). *Genes Genomes Genet.* **5**, 449-465.

Marone, D., Russo, M.A., Laidò, G., de Vita, P., Papa, R., Blanco, A., Gadaleta, A., Rubiales, D. and Mastrangelo, A.M. (2013) Genetic basis of qualitative and quantitative resistance to powdery mildew in wheat: from consensus regions to candidate genes. *BMC Genomics* **4,** 562.

Srinivasachary, Gosman, N., Steed, A., Simmonds, J., Leverington-Waite, M., Wang, Y., Snape, J. and Nicholson, P. (2008) Susceptibility to Fusarium head blight is associated with the *Rht-D1b* semi-dwarfing allele in wheat. *Theor. Appl. Genet.* **116,** 1145-1153.

Wang, G.M., Leonard, J.M., Ross, A.S., Peterson, C.J., Zemetra, R.S., Campbell, K.G., and Riera-Lizarazu, O. (2012) Identification of genetic factors controlling kernel hardness and related traits in a recombinant inbred population derived from a soft X 'extra-soft' wheat (*Triticum aestivum* L.) cross. *Theor. Appl. Genet.* **124,** 207-221.

Yu, H., Deng, Z., Xiang, C. and Tian, J. (2014) Analysis of diversity and linkage disequilibrium mapping of agronomic traits on B-genome of wheat. *J. Genomics* **2,** 20-30.
