# Supplementary material for: Sources of resistance and susceptibility to Septoria tritici blotch of wheat
Source: Mol Plant Pathol. 2016 Oct 20;18(2):276–92. doi: 10.1111/mpp.12482 (PMC5297993; doi:10.1111/mpp.12482)
Supplement: Supplementary file 3 — Table S2 One hundred and twenty‐nine simple‐sequence repeat (SSR) loci tested, with estimated position and number of alleles detected and loci chosen for analysis of population structure. [file MPP-18-276-s003.pdf]

L.S Arraiano & J.K.M. Brown, 2016. Sources of resistance and susceptibility to Septoria tritici blotch of wheat. ***Molecular Plant Pathology.*** **DOI : 10.1111/mpp.12482.**

Supporting Information

Table S2 129 SSR loci tested, with estimated position and number of alleles detected and loci chosen for analysis of population structure.

Chrom.	Locus	Position[1] (cM)	Source of map position	Pop. structure	Allele Number Total	Common[2]	Effective
1A	*Xgwm136*	13.9	NBP		21	4	3.28
	Xwmc24	37.4	NBP	X	6	3	2.16
	Xpsp3027	50.0	NBP		5	3	2.95
	psp3001a	50.0	NBP		3	2	1.91
	Xgwm357	51.3	NBP		4	3	1.96
	Xgwm135	54.4	NBP	X	6	3	1.75
1B	*Xpsp3000*	1.3	NBP	X	8	3	1.41
	Xgwm18	49.0	NBP		5	3	2.87
	Xgwm11	57.0	NBP		7	3	2.28
	Xgwm274	76.8	NBP		7	4	3.24
	Xpsp3100	93.3	NBP		12	4	2.80
	Xwmc44	100.0	NBP	X	7	5	1.98
1D	*Xbarc152*	25.1	NBP	X	9	5	3.28
	Xgwm106	27.8	NBP		2	2	1.93
	Xgwm337	46.0	Röder		9	4	2.43
	Xgwm642	81.5	NBP		4	2	1.57
	Xgdm111	136.6	NBP	X	4	4	1.61
2A	*Xpsp3029a*	24.0	Stephenson		3	2	1.90
	Xbarc124	15.4	NBP	X	4	3	1.67
	Xgwm636	17.7	NBP		9	7	5.53
	Xwmc177	22.0	NBP		9	4	3.06
	Xgwm95	56.1	NBP		5	3	2.11
	Xpsp3088	62.0	NBP		6	2	1.96
	Xgwm445	71.3	NBP	X	4	2	1.31
2B	*Xwmc154*	35.1	NBP	X	5	4	2.36
	Xgwm257	46.9,58.2	NBP		4	3	1.96
	Xgwm388	58,82.4	NBP		6	3	2.84
	Xbarc167	72.2	NBP		3	2	1.12
	Xgwm526a	98.6	NBP, Perry	X	3	3	2.59
	Xgwm526b	98.6	NBP, Perry		5	3	2.20
2D	*Xbarc124*	7.5	NBP	X	6	4	3.10
	Xbarc95	17.8	Liu		3	3	1.73
	Xgwm261a	23.2	Somers		3	2	1.28
	Xgwm261b	23.2	Somers		3	3	2.95
	Xgwm261c	23.2	Somers		4	2	1.54
	Xgwm455	26.3	NBP		3	2	1.42
	Xgwm539	71.2	NBP		9	5	2.93
	Xgwm349	104.3,117.4	NBP	X	10	4	2.90

Chrom.	Locus	Position[1] (cM)	Source of map position	Pop. structure	Allele Number		
					Total	Common[2]	Effective
3A	*Xbarc12*	25.3	NBP		10	4	2.37
	Xgwm369	35.9	NBP	X	10	2	1.42
	Xgwm674	45.6	Somers		4	3	1.45
	Xgwm2	72.0	NBP		4	3	1.39
	Xbarc45	73.0	NBP		5	4	2.36
	Xpsp3047	94.0	NBP		4	2	1.99
	Xgwm155	118.4	NBP	X	7	3	2.02
3B	*Xgwm389*	9.9	NBP	X	11	6	3.46
	Xwmc54	23.0	NBP		8	4	2.07
	Xgwm285	52.8	NBP	X	6	4	2.06
	Xbarc164	54.7	NBP		6	3	2.51
	Xwmc56	81.8	Pánková		6	4	2.69
	Xpsp3001b	97.2	NBP		5	4	2.90
3D	*Xgwm161*	4,25.5	NBP	X	2	2	1.66
	Xbarc8	38.0	NBP		9	5	3.68
	Xgwm456	64.1	NBP		6	4	3.03
	Xgwm3	97.4	NBP	X	4	3	2.21
	Xgwm383	81.9,98.9	NBP		5	3	2.19
4A	*Xbarc106*	37.7	NBP	X	5	2	1.24
	Xgwm601	47.1	NBP		6	2	1.28
	Xwmc246	62.0	NBP		4	2	1.36
	Xgwm610	50.6,70.8	NBP		3	2	1.27
	Xdupw4	122.5	Quarrie		5	4	2.56
	Xbarc184	175.6	NBP	X	4	3	2.64
4B	*Xgwm165*	11.8	NBP	X	8	3	1.50
	Xgwm107	30.9	NBP		7	3	2.07
	Xgwm368	35.6	NBP		5	3	2.76
	Xpsp3078	35.6	NBP		5	4	1.73
	Xbarc163	43.4	NBP	X	5	3	2.75
4D	*Xwmc285*	0.0	NBP	X	4	4	3.21
	Xpsp3007	0.3	Stephenson	X	6	3	2.51
	Xgdm129	41.6	Pestsova		3	2	1.62
	Xgwm165	47.1,69.7	NBP		3	2	1.95
	Xpsp3103a	47.4	NBP		2	2	1.16
	Xwmc331	60.4	NBP		3	3	1.71
5A	*Xgwm205a*	7.44, 39.33	NBP		6	4	1.62
	Xgwm304	24.0	NBP	X	10	4	2.53
	Xgwm129	27.3	NBP		6	5	2.44
	Xgwm186	52.6	NBP		6	4	3.13
	Xbarc56	60.7	Somers		4	2	1.95
	Xgwm291a	105.2	NBP	X	3	3	1.59
	Xgwm291b	105.2	Griffiths		6	3	1.71
5B	*Xgwm234*	20.6	NBP	X	8	7	5.15
	Xwmc73	29.1	NBP		5	3	2.30
	Xgwm335	51.0	NBP		9	3	2.33
	Xbarc140	111.7	NBP	X	3	3	1.41

Chrom.	Locus	Position[1] (cM)	Source of map position	Pop. structure	Allele Number		
					Total	Common[2]	Effective
5D	*Xgwm159*	27.5	Somers		2	2	1.69
	Xgwm190	5.2	NBP	X	7	4	3.59
	Xgwm205b	8.7	NBP		4	3	1.51
	Xbarc143	16.4	NBP		4	3	1.47
	Xgwm583	44.5	NBP		4	4	3.53
	Xgwm292	69.3	NBP		7	3	2.52
	Xbarc110	82.9	NBP	X	7	4	2.21
6A	*Xgwm334a*	28.1	NBP	X	6	3	2.31
	Xgwm334b	28.1	Peng		8	4	2.51
	Xpsp3152	80.7	NBP		12	5	3.48
	Xbarc107	89.5	NBP		4	2	2.01
	Xpsp3029	95.1	NBP		2	2	1.78
	Xgwm570	117.6	NBP	X	6	4	2.68
	Xpsp3029b	122.7	Quarrie		9	5	3.76
	Xpsp3071	122.7	Quarrie		8	4	3.34
	Xdupw167	161.2	Peleg		4	3	2.52
6B	*Xpsp3131*	7.0	Stephenson	X	3	2	1.14
	Xpsp3009	95.7	NBP		5	2	1.34
	Xwmc105	114.2	NBP		11	4	3.15
	Xgwm626	129.3	NBP		2	2	1.24
	Xgwm219	157.0	NBP	X	11	6	2.66
6D	*Xpsp3200*	6.0	NBP	X	5	3	1.80
	Xgwm469	39.0	NBP		7	3	1.48
	Xbarc96	91.7	Somers		3	2	1.84
	Xbarc175	103.4	NBP	X	3	2	1.67
	Xgdm98	202.8	NBP		4	2	1.19
7A	*Xgwm130*	26.0	NBP		5	2	1.86
	Xwmc168	26.0	Somers	X	4	3	2.20
	Xbarc108	71.1	Somers		6	4	2.99
	Xbarc29	80.7	Song		4	3	2.36
	Xpsp3001c	207.0	NBP	X	2	2	1.93
	Xwmc346	269.7	Somers		5	4	1.87
7B	*Xgwm46*	0.0,41	NBP	X	13	3	1.87
	Xbarc72	42.0	NBP		3	3	2.08
	Xgwm274	58.4	NBP		4	2	1.79
	Xpsp3033	75.6	NBP		6	2	1.40
	Xgwm577	105.0	NBP	X	15	9	8.11
7D	*Xgwm130*	58.9	Somers	X	4	2	1.36
	Xgwm295	65.5	NBP		4	4	2.32
	Xbarc125	95.4	NBP		5	3	2.55
	Xbarc214	110.6	NBP		6	4	2.13
	Xgwm44	103.7	NBP		6	5	3.51
	Xpsp3113	124.0	NBP		3	3	2.84
	Xbarc76	226.5	NBP	X	4	4	3.68
Mean					5.52	3.28	2.31

[1] Note that the map of Maccaferri *et al.* (2015) was not used here because it does not include the *Xpsp* SSR loci.

[2] Common alleles are those present in five or more lines tested.

REFERENCES

Griffiths, S., Simmonds, J., Leverington, M., Wang, Y., Fish, L., Sayers, L., Alibert, L., Orford, S., Wingen, L., Herry, L., Faure, S., Laurie, D., Bilham, L. and Snape, J. (2009) Meta-QTL analysis of the genetic control of ear emergence in elite European winter wheat germplasm. *Theor. Appl. Genet.* **119,** 383–395.

Liu, Z.H., Anderson, J.A., Hu, J., Friesen, T.L., Rasmussen, J.B. and Faris, J.D. (2005) A wheat intervarietal genetic linkage map based on microsatellite and target region amplified polymorphism markers and its utility for detecting quantitative trait loci. *Theor. Appl. Genet.* **111,** 782-794.

Maccaferri, M., Zhang, J., Bulli, P., Abate, Z., Chao, S., Cantu, D., Bossolini, E., Chen, X., Pumphrey, M. and Dubcovsky, J. (2015) A genome-wide association study of resistance to stripe rust (*Puccinia striiformis* f.sp. *tritici*) in a worldwide collection of hexaploid spring wheat (*Triticum aestivum* L.). *Genes Genomes Genet.* **5**, 449-465.

National Bioresource Project: *Komugi Wheat Genetic Resources Database: Composite Wheat Map.* http://shigen.lab.nig.ac.jp/wheat/komugi/maps/markerMap.jsp.

Pánková, K., Milec, Z., Simmonds, J., Leverington-Waite, M., Fish, L. and Snape, J.W. (2008) Genetic mapping of a new flowering time gene on chromosome 3B of wheat. *Euphytica* **164,** 779-787.

Peleg, Z., Saranga, Y., Suprunova, T., Ronin, Y., Röder, M.S., Kilian, A., Korol, A.B. and Fahima, T. (2008) High-density genetic map of durum wheat × wild emmer wheat based on SSR and DArT markers. Theor. Appl. Genet. **117:** 103-115.

Peng, J., Korol, A.B., Fahima, T., Röder, M.S., Ronin, Y.I., Li, Y.C. and Nevo, E. (2000) Molecular genetic maps in wild emmer wheat, *Triticum dicoccoides*: genome-wide coverage, massive negative interference, and putative quasi-linkage. *Genome Res.* **10,** 1509-1531.

Perry, D.J. (2004) Identification of Canadian durum wheat varieties using a single PCR. *Theor. Appl. Genet.* **109,** 55–61.

Pestsova, E., Ganal, M.W. and Röder, M.S. (2000) Isolation and mapping of microsatellite markers specific for the D genome of bread wheat. *Genome* **43**, 689-697.

Quarrie, S.A., Steed, A., Calestani, C., Semikhodskii, A., Lebreton, C., Chinoy, C., Steele, N., Pljevljakusić, D., Waterman, E., Weyen, J., Schondelmaier, J., Habash, D.Z., Farmer, P., Saker, L., Clarkson, D.T., Abugalieva, A., Yessimbekova, M., Turuspekov, Y., Abugalieva, S., Tuberosa, R., Sanguineti, M.C., Hollington, P.A., Aragués, R., Royo, A. and Dodig, D. (2005) A high-density genetic map of hexaploid wheat (*Triticum aestivum* L.) from the cross Chinese Spring X SQ1 and its use to compare QTLs for grain yield across a range of environments. *Theor. Appl. Genet.* **110,** 865-880.

Somers, D.J., Isaac, P. and Edwards, K. (2004) A high-density microsatellite consensus map for bread wheat (*Triticum aestivum* L.). *Theor. Appl. Genet.* **109**, 1105-1114.

Song, Q.J., Shi, J.R., Singh, S., Fickus, E.W., Costa, J.M., Lewis, J., Gill, B.S. Ward, R and Cregan, P.B. (2005) Development and mapping of microsatellite (SSR) markers in wheat. *Theor. Appl. Genet.* **110**, 550-560.

Stephenson, P., Bryan, G., Kirby, J., Collins, A., Devos, K., Busso., C. and Gale, M. (1998) Fifty new microsatellite loci for the wheat genetic map. *Theor. Appl. Genet.* **97,** 946-949.
